# Long‐Term Survival Obtained by Repeated Cytoreductive Surgery and S‐1 Plus Cisplatin Chemotherapy at Each Instance of Disease Progression in a Patient With Metastatic Urachal Carcinoma: A Case Report

**DOI:** 10.1002/cnr2.70317

**Published:** 2025-08-18

**Authors:** Masayasu Urushibara, Daisuke Kato, Taisuke Okumura, Akihiro Kojima, Yuichiro Kato, Takeshi Shirakawa, Yohei Shimizu, Tsunehiro Nenohi, Yuki Matsumoto, Noriyuki Matsutani, Tatsuya Aso, Mikiko Takahashi, Kazuhiro Ishizaka, Minato Yokoyama

**Affiliations:** ^1^ Department of Urology Teikyo University Hospital, Mizonokuchi Kawasaki City Japan; ^2^ Department of Urology Nissan Tamagawa Hospital Tokyo Japan; ^3^ Department of Surgery Teikyo University Hospital, Mizonokuchi Kawasaki City Japan; ^4^ Department of Diagnostic Pathology Teikyo University Hospital, Mizonokuchi Kawasaki City Japan

**Keywords:** chemotherapy, long survival, metastasectomy, metastasis, multimodality treatment, urachal carcinoma

## Abstract

**Background:**

Urachal carcinoma (URC) is a rare tumor of the urinary bladder, of which the histology usually resembles that of colorectal adenocarcinoma. Achievement of cure in patients with metastatic URC is difficult, and the survival rate of these patients has remained unsatisfactory despite various efforts.

**Case:**

A 74‐year‐old female patient presented to us complaining of gross hematuria. Abdominal and thoracic computed tomography revealed a mass in the dome of the bladder with a single lung nodule. The two tumors, which were resected by partial cystectomy and video‐assisted thoracic surgery, respectively, were diagnosed by postoperative histopathology as adenocarcinomas. Subsequent to the surgeries, bilateral ovarian metastases and another lung metastasis, which appeared metachronously, were also resected. The repeated cytoreductive surgery combined with administration of S‐1 plus cisplatin chemotherapy at each instance of disease progression eventually yielded a durable progression‐free survival; even at 5 years after the initial therapy, the patient remained asymptomatic with no limitation of activities despite the failure to achieve “cure”.

**Conclusion:**

Not only some degree of sensitivity of the tumor to chemotherapy, but also the repeated cytoreductive surgeries might allow prolonged survival with a good quality of life in elderly patients with metastatic URC, even in the absence of cure and failure of genetic testing to suggest any potentially effective second‐line drugs. To improve the survival of patients with metastatic URC, complementary therapy suggested by the results of genomic profiling may be necessary along with other multimodality therapy, including sequential metastasectomy and chemotherapy.

**Abbreviations**
CDDP: cisplatin
CEA: carcinoembryonic antigen
CR: complete remission
CRC: colorectal adenocarcinoma
CSS: cancer‐specific survival
CT: computed tomography
dMMR: deficiency of MMR
MSI: microsatellite instability
ORR: overall response rate
OS: overall survival
PET: positron emission tomography
PR: partial response
QOL: quality of life
SD: stable disease
URC: urachal carcinoma
VATS: video‐assisted thoracic surgery

## 1 Introduction

Urachal carcinoma (URC) is a rare cancer accounting for < 1% of all bladder cancers that arises from the urachal remnant [1, 2]. Chemotherapy is the treatment mainstay in patients with metastatic URC, as this is systemic disease. However, despite various therapeutic efforts aimed mainly at cure, the reported 5‐year survival rate of patients with metastatic URC remains dismal at about 10%–20% [3, 4]. Although targeted therapies and immune checkpoint inhibitor therapy have been used in patients with metastatic URC, their roles in the treatment of this cancer remain to be clearly delineated [5, 6]. URC often histologically resembles colorectal cancer (CRC); the genetic profile of URC also more closely resembles that of CRC than the genetic profile of urothelial carcinoma, although genomic profiling often fails to offer a selection of suitable target drugs for treatment [7, 8]. Some authors have noted that metastasectomy should be performed only in limited cases of metastatic URC [9, 10]. Therefore, it would seem that complete resection and/or complete remission (CR) after chemotherapy is necessary to achieve long‐term survival [11]. Currently, there is the concern that neither long‐term survival nor a quality of life (QOL) can be obtained with further therapies in the absence of complete resection and/or CR to chemotherapy and failure of genomic testing to suggest potentially useful drugs. Herein, we report a rare case in which long‐term survival with a good QOL was obtained despite failure of cure in an elderly patient with incurable metastatic URC by repeated metastasectomy and administration of chemotherapy at every instance of disease progression.

## 2 Case

### 2.1 Patient Information With Primary Concerns and Symptoms of the Patient With History and Relevant Interventions and Their Outcome

**FIGURE 1:**
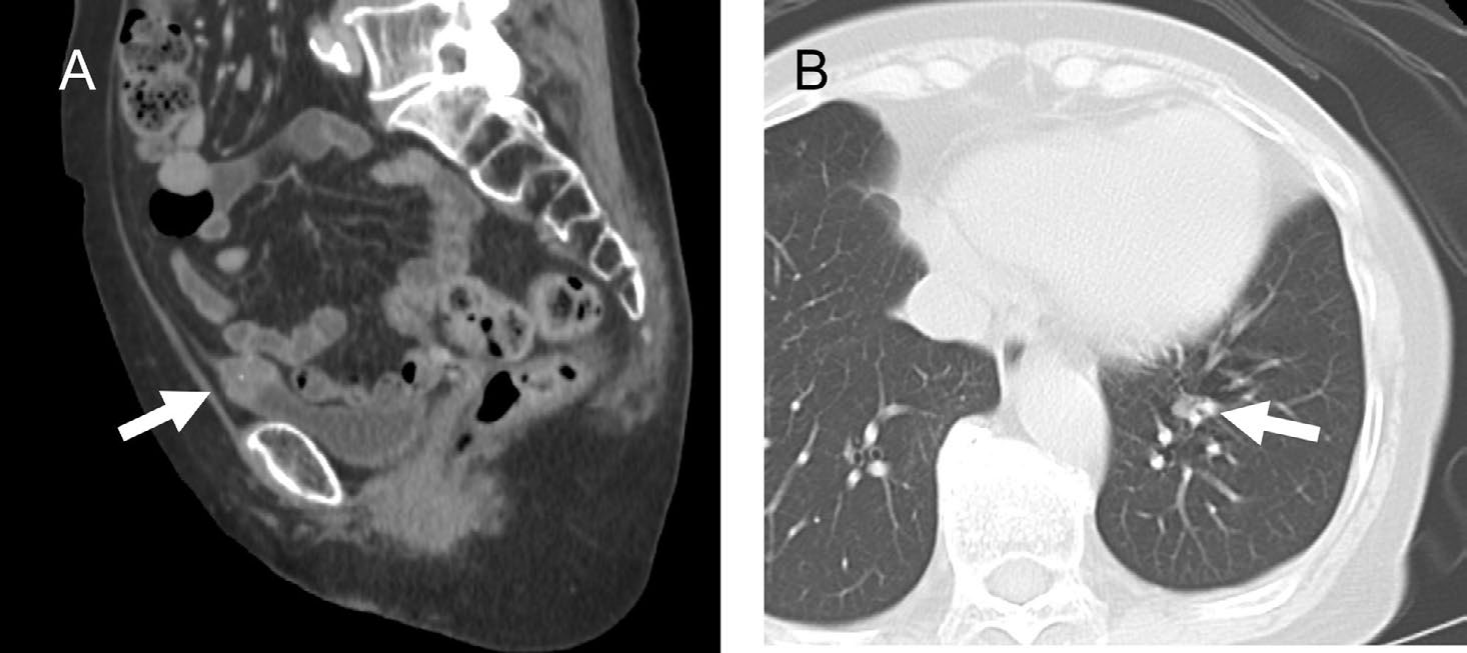
Computed tomographic image showing (A) a mass in dome of the bladder and (B) a nodule in the left lung.

**FIGURE 2:**
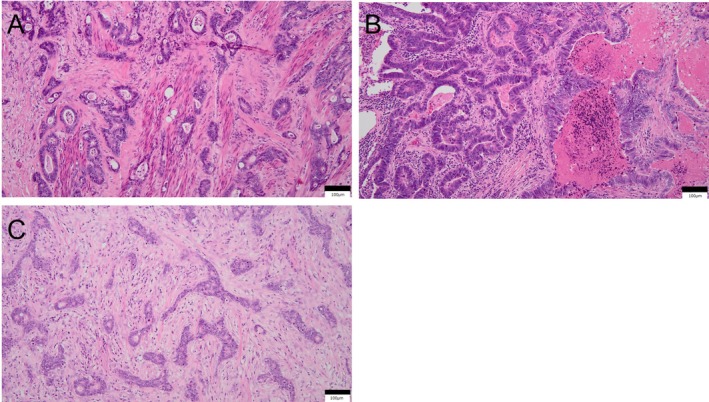
Histopathological examination of (A) the bladder specimen after partial cystectomy, (B) lung specimen after the first video‐assisted thoracic surgery, and (C) ovarian specimen after salpingo‐oophorectomy revealed moderate to well‐differentiated adenocarcinoma (hematoxylin and eosin stain, 100×) at all three sites.

A 74‐year‐old female patient visited our hospital, Teikyo University Hospital, Mizonokuchi, in March 2019 with a 2‐week history of gross hematuria. Her past medical history included bilateral ovarian cysts for which she had been under monitoring by a gynecologist for the past 10 years, subarachnoid hemorrhage, hypertension, sleep disorder, and urticaria. The findings of urinary cytology were categorized as Class III. The Karnofsky performance status was rated as 90. Abdominal and thoracic computed tomography (CT) revealed a mass in the bladder dome, along with a nodule measuring about 5 mm in diameter in the left lung (Figure 1A,B). Cystoscopy revealed a single irregular recessed tumor. The serum carcinoembryonic antigen (CEA) level was elevated to 21.7 ng/mL (normal range, ≤ 5 ng/mL), but we considered it unnecessary to perform an examination of the gastroenterological tract to identify the cause of the elevated serum CEA. We performed transurethral resection of the bladder tumor in April 2019, and postoperative histopathology revealed adenocarcinoma. We then performed CT‐guided biopsy of the lung tumor to exclude primary lung carcinoma, which revealed histological features compatible with metastasis from the URC. Based on the findings, we diagnosed the patient as having URC, clinical stage IVB according to Sheldon's classification. Therefore, in July 2019, we performed complete resection of the residual bladder tumor by partial cystectomy and resection of the urachal remnant (Figure 2A). A month later, in August 2019, we performed video‐assisted thoracic surgery (VATS) to resect the lung metastasis (Figure 2B). Thereafter, in October 2019, we initiated the patient on 5 cycles of combined chemotherapy with S‐1 plus cisplatin (CDDP) (S‐1: 40–60 mg, depending on the patient's body surface area, administered per os twice daily, 3 weeks on and 2 weeks off; CDDP: 60 mg/m^2^ by intravenous injection [iv] on Day 8) [12].

### 2.2 Important Clinical Findings and Therapeutic Interventions After the Initial Therapy

**FIGURE 3:**
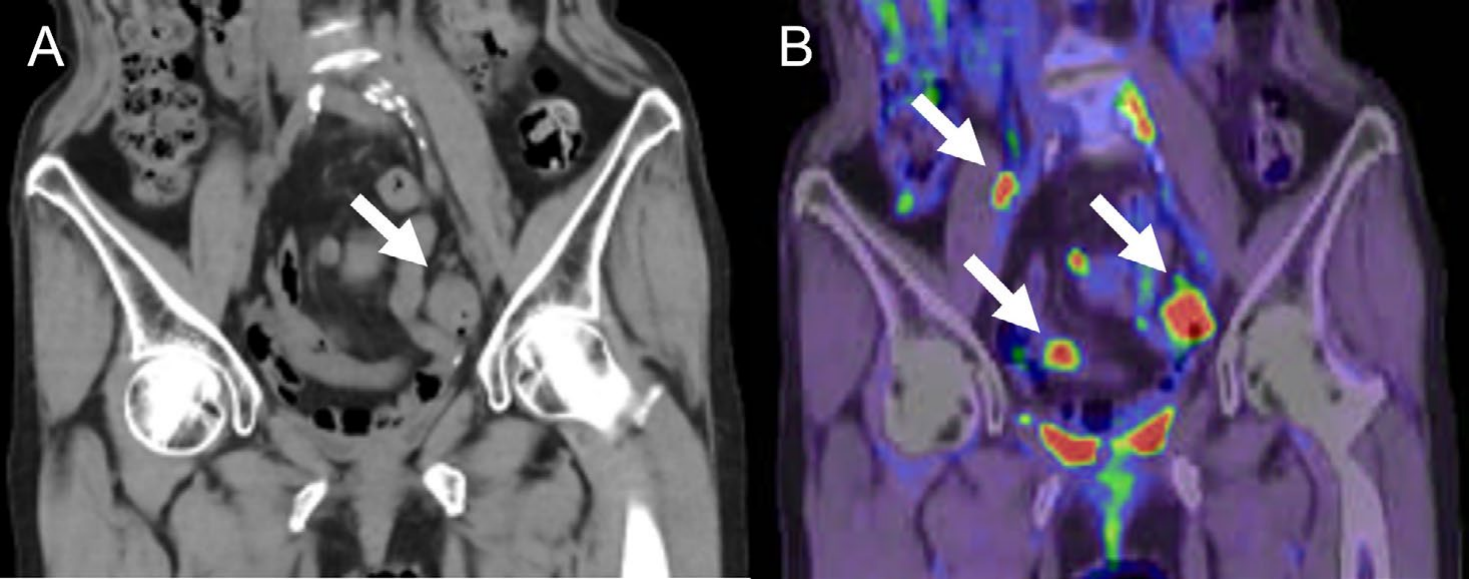
Radiographic findings after the second video‐assisted thoracic surgery. (A) A plain, non‐contrast pelvic computed tomography (CT) demonstrated an enlarged left common iliac lymph node swelling, while (B) fluorodeoxyglucose positron emission tomography/CT revealed multiple hot spots in the pelvic area.

**FIGURE 4:**
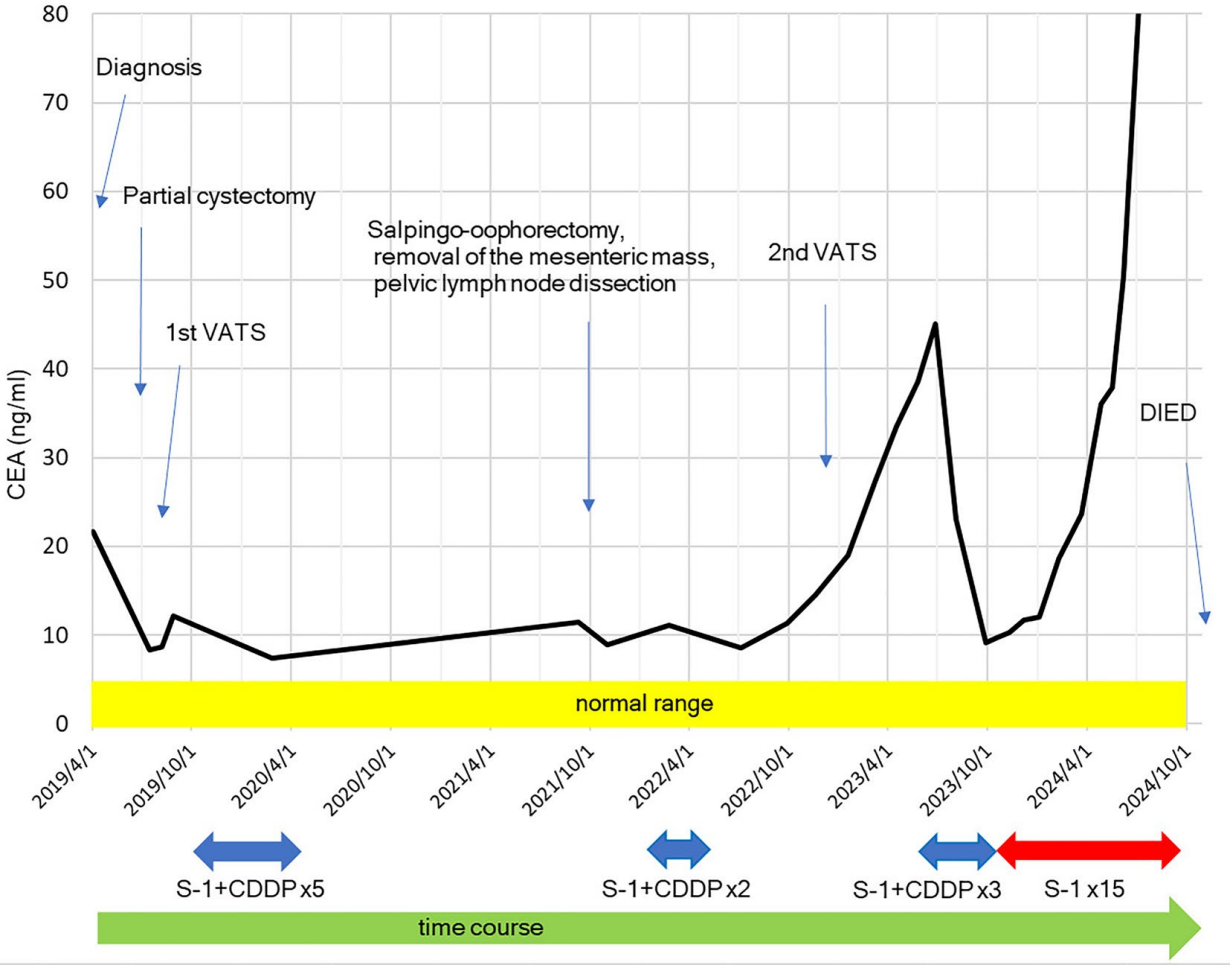
Time‐course of changes in the serum carcinoembryonic antigen levels.

Follow‐up CT performed every 3 months revealed enlargement of the left ovary, a mesenteric nodule, and enlarged right external iliac and left common iliac lymph nodes at 18 months after the initial therapy, with a subsequent CT in September 2021 showing progressive enlargement in the sizes of the lymph nodes. In October 2021, as we could not rule out the presence of a contralateral ovarian metastasis intraoperatively during surgery for the enlarged ovary, we performed bilateral salpingo‐oophorectomy with removal of the mass in the mesentery of the small intestine and the enlarged right external iliac lymph node to confirm the presence of metastasis. We left the enlarged left common iliac lymph node intact as it was adherent to an iliac vessel and planned to administer chemotherapy after the surgery. Postoperative histopathology confirmed metastases from the URC (Figure 2C); there was no evidence of microsatellite instability. After discussing further treatment options with the patient, in February 2022, 4 months after the surgery, we started the patient again on S‐1 plus CDDP therapy. Follow‐up chest CT performed 1 month after the re‐start of chemotherapy revealed a 5‐mm metastatic nodule in the superior lingular segment of the left lung. However, the chemotherapy needed to be interrupted after 2 courses as the patient sustained a lumbar compression fracture caused by a fall resulting from dizziness developing just after a contrast CT. Thereafter, the serum CEA level increased to 11.3 ng/mL, and by the end of 6 months, in September 2022, the single metastatic nodule in the left lung had grown to 7 mm in diameter, presumably because of the treatment interruption. In December 2022, we performed a second VATS for the left lung nodule. However, even after the surgery, the serum CEA level continued to increase (38.6 ng/mL), and a fluorodeoxyglucose (FDG)‐positron emission tomography (PET)/CT revealed accumulation in multiple intrapelvic lymph nodes and/or intraperitoneal dissemination, although an abdominal CT in May 2023 revealed that the size of the enlarged single left common iliac lymph node that was left behind during the first surgery had remained unchanged for 18 months (Figure 3A,B). We considered that the cancer still remained sensitive to S‐1 plus CDDP therapy as there was no apparent disease progression during the previous courses of therapy and that the disease had begun to progress only after the treatment interruption (Figure 4). Therefore, in June 2023, we initiated the patient on a further three courses of S‐1 plus CDDP. After every CDDP dose, the patient developed the tolerable adverse event of generalized fatigue and nausea lasting for about a week. In October 2023, when the total CDDP dose used had exceeded 500 mg, we discontinued the CDDP and continued the patient on S‐1 alone (40 mg twice daily, 2 weeks on and 1 week off). With S‐1‐alone therapy in the absence of CDDP, the serum CEA level began to increase steadily. However, a follow‐up PET/CT in December 2023 revealed no apparent disease progression. Comprehensive genomic profiling revealed *TP53* and *RNF43* alterations in the lung metastasis resected at the second VATS (FoundationOne CDx next‐generation sequencing test), but the tumor mutation burden was low. However, we could not identify any effective drugs. In addition, since the patient also began to complain of generalized fatigue that prevented her from doing housework after each dose of S‐1, the dose of S‐1 was decreased to 20 mg per day. Thereafter, the serum CEA level continued to increase (18.7 ng/mL), and a chest CT repeated in February 2024 revealed three lung metastases involving both lungs, each measuring about 8 mm in diameter. The patient sought a second opinion again in April 2024 and was told that further chemotherapies for the lung metastases and carcinoperitonitis cannot be recommended because no definitely effective chemotherapies for metastatic URC had been identified yet, and moreover, that the elderly patient, almost 80 years old by this time, was at a high risk of developing adverse events with further chemotherapy. Based on the advice, the patient and her husband decided against receiving further therapy, except for the daily S‐1 at 20 mg/day. Thereafter, while her QOL was maintained, the serum CEA increased to 97.6 ng/mL, and abdominal CT in July 2024 revealed a single liver metastasis, about 2 cm in diameter. Magnetic resonance imaging of the brain in August 2024 did not reveal any brain metastasis.

### 2.3 Details of Further Follow‐Up of the Patient: Last Follow‐Up Visit, Patient's Clinical Condition, Quality of Life With Any Other Significant Features

S‐1 was continued for a total of 15 courses, but discontinued in September 2024 as the patient developed abdominal symptoms associated with stenosis of the sigmoid colon. Histological examination of endoscopic biopsy specimens obtained from the colon showed features of adenocarcinoma resembling those of URC. The patient developed severe anemia due to persistent gastrointestinal bleeding. The serum CEA increased to 467.0 ng/mL; the patient died in October 2024. No autopsy was performed.

## 3 Discussion

The treatment mainstay for metastatic URC is chemotherapy. However, according to systematic reviews, the overall response rate (ORR) of metastatic URC to chemotherapy remains poor, at about 30%–40% [10, 13, 14]. Histologically, more than 80% of URCs are adenocarcinomas. These tumors also bear genetic resemblance (e.g., KRAS, BRAF mutations) to CRC [8, 15]. Therefore, chemotherapeutic regimens such as FOLFOX that are used to treat CRC have also been applied to patients with metastatic URC [4, 10, 16]. On the other hand, combined therapy with S‐1 plus CDDP, a well‐acknowledged effective regimen for patients with advanced gastric cancer [17], has also been used for treating patients with metastatic URC for the following reasons: [5, 12] S‐1, a prodrug of 5‐FU, is not only more effective, but is also associated with fewer adverse events than 5‐FU; furthermore, urologists have been using CDDP for many years to treat urothelial carcinoma and testicular tumors and consider it easier and safer to use CDDP rather than any of the other drugs that are conventionally used to treat CRC.

Although effective chemotherapy combinations have been employed, the median overall survival (OS) from commencement of chemotherapy to the development of metastasis somehow reached about 2 years [10, 14]. According to one report, after propensity score matching, the OS and cancer‐specific survival (CSS) of patients who had received chemotherapy were higher than those of patients who had not received chemotherapy (OS: median 16 vs. 3 months; CSS: median 17 vs. 4 months) [18]. According to another study, chemotherapy offered a statistically significant improvement in the 12‐month survival as compared with surgical treatment in patients with metastatic URC (*p* = 0.035) [19], whereas no significant differences were observed at 6, 24, 36, 48, and 60 months between patients treated by metastasectomy and those treated by chemotherapy. Yet another study reported that chemotherapy provided no apparent survival benefit (HR 0.785, 95% CI 0.37–1.65) in patients with metastatic URC [20]. Cautious interpretation of these results is needed because of the variety of chemotherapy regimens used to treat the patients with metastatic URC with heterogeneous clinical backgrounds in these studies. However, at the least, these results suggest that chemotherapy alone can hardly be expected to provide long‐term survival benefit in most patients with metastatic URC, although sensitivity to chemotherapy was noted in patients showing long‐term survival [21, 22].

Some authors have noted that metastasectomy should be performed only in limited cases of metastatic URC [9, 10]. Metastasectomy for metastatic URC should be cautiously performed after taking multiple factors into account, including the general condition of the patient, determination that the metastatic site(s) is surgically resectable, sites of metastasis, number of organs with distant metastases, tumor number and sizes in the metastatic organ, rate of growth of the metastatic tumor, and response to chemotherapy. It would undoubtedly be ideal if complete surgical resection of recurrent tumors could be assigned the highest priority for obtaining prolonged survival in patients with metastatic URC [11, 22]. Attention should also be paid to cases that show a good response to chemotherapy. To the best of our knowledge, there is no consensus yet on the indication for metastasectomy in patients with metastatic URC depending on the degree of response to chemotherapy, which can suppress the rate of growth of URC. Therefore, to investigate this association, we reviewed the literature to identify case reports of metastatic URC published in the 21st century, in particular, to weigh the contribution of chemotherapy and/or metastasectomy in prolonging patient survival. We compared the metastatic status and the best response to chemotherapy between patients who survived for over 5 years (long‐term survivors; Table 1) and those who died within 2 years (short‐term survivors; Table 2). In the patients covered by our review, none of the short‐term survivors in whom the best response to chemotherapy was partial response (PR) or stable disease (SD), except for one case, had undergone metastasectomies for metastatic sites that had been treated with chemotherapy. Among the long‐term survivors, there were two cases with repeated metastases in which the best response to chemotherapy was PR or SD. Kawakami reported that a 30‐year‐old patient whose best response to chemotherapy was PR underwent metastasectomy twice and received three kinds of chemotherapies sequentially over a period of about 3 years, and thereafter lived without any evidence of disease recurrence for 8 years [11]. Micheletti reported that in a 51‐year‐old patient, a second intraperitoneal cytoreductive surgery plus hyperthermic intraperitoneal chemotherapy with cisplatin plus mitomycin C after neoadjuvant systemic chemotherapy (FOLFIRI [leucovorin calcium, 5‐FU and irinotecan] and bevacizumab) suppressed the growth of metastatic URC that had earlier been resistant to both the metastasectomy and chemotherapy with XELOX (capecitabine plus oxaliplatin) [28]. It should be kept in mind that not only tumor progression, but also adverse events and complications associated with metastasectomy could worsen the QOL, especially in elderly patients. Although targeted therapy and immune checkpoint inhibitors are expected to play a role in the future in improving the prognosis of patients with metastatic URC, their adverse effects could lower the QOL of the patients. Metastasectomy should also be performed taking into consideration its safety and tolerability. Our elderly patient reported herein tolerated the repeated administrations of chemotherapy using the same drugs and repeated metastasectomies.

**TABLE 1:** Cases of metastatic urachal carcinoma with long‐term survival reported in the 21th century.

Author	Year	Literature	Age	Gender	Period from surgical resection of the primary disease to appearance of metastasis (months)	Local therapy	All metastases	Resected metastases
Kawakami [11]	2001	Urology	30	F	0	PC	Intrapelvic lymph node → lung, ovary	Lung, ovary
Kawakami [11]	2001	Urology	55	M	4	PC	Intrapelvic lymph node → lung	Intrapelvic lymph nodes
Morii [21]	2007	Int J Urol	65	M	6	PC	Ureter → paraaortic lymph node	No
Tatokoro [23]	2008	Int J Urol	64	M	13	RC	Lung	No
Sugarbaker [24]	2008	Tumori	32	F	12	PC	Peritoneum → peritoneum → peritoneum	Peritoneum → peritoneum → peritoneum
Loh [25]	2016	Clin Genitourin Cancer	42	F	36	PC	Lung → local recurrence, colon	No
Yasui [22]	2018	Case Rep Urol	56	M	0	PC	Inguinal lymph nodes, peritoneum, omentum → vermiform appendix, abdominal rectus muscle	Inguinal lymph node → peritoneum, omentum, appendix, rectus abdominis muscle
Somiya [26]	2020	Urol Case Rep	48	M	13	PC	Lung → lung	Lung → lung
Stokkel [27]	2021	Eur Urol Open Sci	55	M	96	PC	Prostatic urethra → urethra → penis, bone	Prostatic urethra → urethra → penis
Mathavan [6]	2023	Clin Genitourin Cancer	73	M	0	RC	Lung → liver, obturator muscle	No
Micheletti [28]	2024	Int J Surg Case Rep	51	F	7	PC	Peritoneum, omentum → peritoneum	Peritoneum, omentum
Present case	NA	NA	74	F	0	PC	Lung → ovary, mesenterium, pelvic lymph nodes → lung → peritoneum → liver	Lung → ovary, mesenterium, pelvic lymph nodes → lung

Author	Metastasis for chemotherapy	Chemotherapy regimen (courses), radiation for metastatic sites	Best response to chemotherapy	Period from diagnosis of metastasis to latest follow‐up (months)	Outcome	Genetic alterations (next‐generation sequencing)
Kawakami [11]	Intrapelvic lymph nodes → lung	5‐FU + ADM + CDDP for adjuvant (5) → 5‐FU + ADM + VP‐16 (2) → 5‐FU + CDDP+interferon‐α for neoadjuvant (NA)	PR	132	AWN	NA
Kawakami [11]	Lung	ADM + MMC + CDDP (3) + radiation (lung 50 Gy) → uracil+tegafur(NA)	CR	over 120	AWN	NA
Morii [21]	Adjuvant → ureter → paraaortic lymph nodes	Epirubicin + MMC + 5‐FU, including adjuvant (more than 2)	CR	60	AWN	NA
Tatokoro [23]	Lung	IFEP (ifosfamide+5‐FU + VP‐16 + CDDP) (4) → radiation (lung 50 Gy)	CR	112	AWN	NA
Sugarbaker [24]	Peritoneum → peritoneum → peritoneum	intraperitoneal MMC + 5‐FU for adjuvant (1) → intraperitoneal CDDP+ADM for adjuvant (1) → intraperitoneal CDDP for adjuvant (1)	PD	120	DFC	NA
Loh [25]	Lung → local recurrence, colon	FOLFIRI (levofolinate calcium+5‐FU + CPT‐11), including adjuvant (more than 13) → capecitabine (NA) → GC (GEM+CDDP) + 5‐FU (NA) → GC (NA) → PTX + MTX + CDDP (NA) → trametinib (NA) → PTX (3)	SD	72	DFC	GNAS R201H, KRAS G12D, TP53 R273H (recurrent bladder tumor)
Yasui [22]	Peritoneum	GC (5) → FOLFIRI (11)	PR	over 71	AWN	NA
Somiya [26]	Adjuvant	XELOX (capecitabine+oxaliplatin) for adjuvant (8)	PD	77	AWN	NA
Stokkel [27]	No	Radiation (prostate 47 Gy)	NA	82	DFC	NA
Mathavan [6]	Lung	Atezolizumab (38) → ipilimumab (3) → nivolumab (3) → ipilimumab +nivolumab (4) → XELOX (10?) → CDDP+PTX (4) → radiation (obturator muscle)	PR	69	DFC	SMAD4, KRAS (liver metastasis)
Micheletti [28]	Peritoneum, omentum	Intraperitoneal CDDP+MMC for adjuvant (1) → XELOX (6) → FOLFIRI+bevacizumab (NA) → intraperitoneal CDDP+MMC for adjuvant (1) → bevacizumab for adjuvant (12)	PR	86	AWN	NA
Present case	Pelvic lymph nodes → peritoneum → liver	S‐1 + CDDP (10) → S‐1 (15)	SD	67	DFC	TP53, RNF43 (recurrent lung metastasis)

**TABLE 2:** Cases of metastatic urachal carcinoma with short‐term survival reported in the 21th century.

Author	Year	Literature	Age	Gender	Period from surgical resection of the primary disease to appearance of metastasis (months)	Local therapy	All metastases	Resected metastases
Kaido [29]	2003	J Clin Neurosci	62	M	24	PC → radiation	Lung → brain → brain	Brain (incomplete resection) → brain (incomplete resection)
La Fianza [30]	2005	Tumori	26	M	0	RC	Intrapelvic lymph node, bone → soft tissue around mandible	No
Idei [31]	2005	No Shinkei Geka [Article in Japanese]	64	F	0	PC	Brain → brain, lung	Brain
McClelland [32]	2006	J Neurooncol	33	F	36	PC	Lung, lymph nodes (lung and paratracheal) → subcutaneous fat of abdomen, brain, bone → carcinomatous meningitis	Lung → brain
Tatokoro [23]	2008	Int J Urol	51	F	9	PC → radiation (50Gy)	Intrapelvic lymph nodes → cervical lymph nodes, brain	No
Tatokoro [23]	2008	Int J Urol	66	M	13	RC	Lung, liver	No
Egevad [33]	2009	Scand J Urol Nephrol	51	M	16	PC	Intrapelvic lymph nodes → peritoneum	No
Elser [34]	2012	Can Urol Assoc J	35	F	5	PC	Lung, intrapelvic lymph nodes → adnexa, peritoneum, omentum	No
Zong [35]	2013	World J Surg Oncol	41	M	6	PC	Liver → ovary, omentum → contralateral ovary → peritoneum	Ovary, omentum (incomplete resection) → contralateral ovary (incomplete resection)
Testa [36]	2014	Rare Tumors	33	F	0	PC	Lung → lung → peritoneum	Lung
Ormeci [37]	2015	Case Rep Radiol	61	M	27	PC	Peritoneum → colon, peritoneum, pleura, pericardial fat, lung	Colon
Paschke [38]	2016	World J Surg Oncol	34	F	30	Radiation (50.4Gy)	Liver	Liver
Yaegashi [39]	2019	Mol Clin Oncol	55	F	0	NA	Peritoneum	No
Stokkel [27]	2021	Eur Urol Open Sci	50	M	11	RC → radiation	Prostatic urethra, penis → liver	Urethra
Murakami [40]	2023	No Shinkei Geka [Article in Japanese]	67	F	0	NA	Brain → brain, lung, liver	Brain
Mathavan [6]	2023	Clin Genitourin Cancer	56	M	0	PC	Brain, lung, liver	No
Bashour [41]	2024	Ann Med Surg (Lond)	71	F	0	No	Liver	No

Author	Metastasis for chemotherapy	Chemotherapy regimen (courses), radiation for metastatic sites	Best response to chemotherapy	Period from diagnosis of metastasis to latest follow‐up (months)	Outcome	Genetic alterations (next‐generation sequencing)
Kaido [29]	NA	*γ*‐knife	NA	over 6	DFC	NA
La Fianza [30]	Intrapelvic lymph nodes, bone	M‐VAC (MTX, VBL, ADM, CDDP) (NA)	SD	22	DFC	NA
Idei [31]	NA	Radiation (brain 60Gy)	NA	17	DFC	NA
McClelland [32]	Lung, lymph nodes (lung and paratracheal) → subcutaneous fat of abdomen, brain, bone	5‐FU + CPT‐11 + bevacizimab (NA) → temozolomide (NA)	PD	12	DFC	NA
Tatokoro [23]	Intrapelvic lymph nodes	IFEP (IFM + 5‐FU + VP‐16 + CDDP) (4)	PR	20	DFC	NA
Tatokoro [23]	Lung, liver	IFEP (2)	PD	7.5	DFC	NA
Egevad [33]	Adjuvant → peritoneum	M‐VAC (4) → eloxatin‐FLV (13)	PD	10	DFC	NA
Elser [34]	Lung, intrapelvic lymph nodes	GC (GEM+CDDP) (NA) → PTX + CBDCA(9) → capecitabine+antiangiogenic agent(6) → CPT‐11 (3) → cyclophosphamide+prednisolone (NA) → XELOX (capecitabine+oxaliplatin) (1)	PD	over 9	DFC	NA
Zong [35]	Adjuvant	PTX + CBDCA (4) → GC (4) → IFO + EPI (4) → docetaxel+oxaliplatin+capecitabine (NA)	SD	over 18	DFC	NA
Testa [36]	Lung → peritoneum	CPT‐11 + capecitabine+oxalipaltin+ (NA) → sunitinib (NA)	SD	8	DFC	NA
Ormeci [37]	No	No	NA	6	DFC	NA
Paschke [38]	Adjuvant	PTX + CBDCA (4) → GEM (3)	PD	14	DFC	NA
Yaegashi [39]	Peritoneum	GC (more than 7)	SD	23	DFC	NA
Stokkel [27]	Liver	XELOX (6) → CPT‐11 (NA)	SD	12	DFC	NA
Murakami [40]	No	No	NA	4.5	DFC	NA
Mathavan [6]	Brain, lung, liver	FOLFOX (leucovorin, 5‐FU, oxaliplatin) (6) for adjuvant→ pembrolizumab (3)	PR	21	DFC	NA
Bashour [41]	Liver	FOLFOX6 (folinic acid, 5‐FU, oxaliplatin) (6) → GemCarbo (GEM+CBDCA) (6) → FOLFIRI‐B (folinic acid, FU, CPT‐11, bevacizumab) (2)	PR	20	DFC	wild type EGFR (liver metastasis)

Genomic profiling has been used to select the appropriate treatments for URC, but its value remains to be fully evaluated. According to limited investigations conducted on small numbers of URC patients, although genetic variations (e.g., KRAS, BRAF mutations) in URC resemble those in CRC, the frequency of genetic alterations in URC is not high [3, 7, 8]. Moreover, even among patients in whom genetic alterations are detected, suitable treatments can be identified only in a limited number of patients. The presence of KRAS and BRAF mutations in CRC is reported to be associated with a poor response to EGFR monoclonal antibodies [7]. A consistent association has been shown between deficiency of MMR (dMMR) and microsatellite instability (MSI), and these could predict the efficacy of PD‐1 inhibitor therapy. However, in the case of URC, there has been very little research, and only a very low frequency of dMMR has been reported [3, 42]. Therefore, in the absence of genomic profiling offering any suitable target drugs, metastasectomy could become an alternative second‐ and/or further‐line therapy. Moreover, while genomic variations could also differ between the primary and metastatic tumors and new genomic alterations could develop after each therapy for repeated metastases, genomic profiling of metastatic tumor specimens resected by metastasectomy could allow selection of suitable drugs [43, 44, 45]. In the limited number of long‐term and short‐term survivors included in our review, genomic profiling was performed; whereas, in most of the long survival cases, different chemotherapies were used to try to meet suitable drugs, respectively. Genomic profiling would be expected to help in the selection of suitable drugs as soon as possible and thereby possibly contribute to improving the survival of patients with metastatic URC that are resistant to various chemotherapies. In the future, to evaluate and consider willing to therapy, the physical, mental, and social status of patients, as well as tumor character and the degree of metastasis each time, genomic tests, chemotherapy, and metastasectomy combined complementarily may be important to offer improved survival with a good QOL in patients with metastatic URC.

There were some limitations of this study. If PET‐CT is performed earlier than it was in our patient, carcinoperitonitis could be detected earlier. Our literature review was based only on a small number of reports of metastatic URC in the 21st century, because of the low frequency of cases of metastatic URC in the population. Moreover, the backgrounds of the patients, the therapies adopted, and the follow‐up periods were heterogeneous.

## 4 Conclusions

Our experience with this case suggests that to obtain long survival with a good QOL even in the absence of cure in elderly patients with incurable metastatic URC, not only some degree of chemotherapy sensitivity, but also cytoreductive surgery before the disease becomes far advanced is necessary; even if genomic profiling of the tumors fails to offer a selection of suitable drugs. Complementary use of target drugs suggested by genomic profiling with multimodality therapy, including metastasectomy and chemotherapy, in properly selected cases could be expected to improve survival in patients with metastatic URC.

## Author Contributions

Conception and design of study: Masayasu Urushibara, Daisuke Kato, Taisuke Okumura, Akihiro Kojima, Yuichiro Kato, Minato Yokoyama. Data analysis and/or interpretation: Masayasu Urushibara, Daisuke Kato, Taisuke Okumura, Takeshi Shirakawa, Yohei Shimizu, Noriyuki Matsutani, Minato Yokoyama. Histological examination: Tatsuya Aso, Mikiko Takahashi. Drafting of the manuscript and/or critical revision: Masayasu Urushibara, Daisuke Kato, Taisuke Okumura, Tsunehiro Nenohi, Yuki Matsumoto, Noriyuki Matsutani, Kazuhiro Ishizaka, Minato Yokoyama.

## Ethics Statement

Our institution does not require ethical approval and consent to participate for reporting retrospective fully‐anonymized case reports. There were no other ethical concerns in relation to this article.

## Consent

Informed consent was orally received from the patient for publication of the anonymized information and accompanying images in this article; it was voluntarily written by the authors in the medical record when the patient was alive, in case something happens.

## Conflicts of Interest

The authors declare no conflicts of interest.

## Data Availability

Data sharing not applicable to this article as no datasets were generated or analysed during the current study.
